# Attenuation of PI3K-Akt-mTOR Pathway to Reduce Cancer Stemness on Chemoresistant Lung Cancer Cells by Shikonin and Synergy with BEZ235 Inhibitor

**DOI:** 10.3390/ijms25010616

**Published:** 2024-01-03

**Authors:** Yen-Hsiang Huang, Ling-Yen Chiu, Jeng-Sen Tseng, Kuo-Hsuan Hsu, Chang-Han Chen, Gwo-Tarng Sheu, Tsung-Ying Yang

**Affiliations:** 1 Division of Chest Medicine, Department of Internal Medicine, Taichung Veterans General Hospital, Taichung 407, Taiwan; waynehuang0622@gmail.com (Y.-H.H.); iang0810@gmail.com (L.-Y.C.); tzeng64@gmail.com (J.-S.T.); 2 Faculty of Medicine, School of Medicine, National Yang Ming Chiao Tung University, Taipei 112, Taiwan; 3 Department of Post-Baccalaureate Medicine, College of Medicine, National Chung Hsing University, Taichung 402, Taiwan; 4 Division of Critical Care and Respiratory Therapy, Department of Internal Medicine, Taichung Veterans General Hospital, Taichung 407, Taiwan; vghryan@gmail.com; 5 Department of Applied Chemistry, Graduate Institute of Biomedicine and Biomedical Technology, National Chi Nan University, Nantou 545, Taiwan; changhan155@hotmail.com; 6 Department of Medical Research, Taichung Veterans General Hospital, Taichung 407, Taiwan; 7 Institute of Medicine, Chung Shan Medical University, Taichung 402, Taiwan; 8 Department of Medical Oncology and Chest Medicine, Chung Shan Medical University Hospital, Taichung 402, Taiwan; 9 Department of Life Sciences, National Chung Hsing University, Taichung 402, Taiwan

**Keywords:** shikonin, chemoresistance, lung cancer stem cells, PI3K/Akt/mTOR pathway, BEZ235, synergy

## Abstract

Lung cancer is considered the number one cause of cancer-related deaths worldwide. Although current treatments initially reduce the lung cancer burden, relapse occurs in most cases; the major causes of mortality are drug resistance and cancer stemness. Recent investigations have provided evidence that shikonin generates various bioactivities related to the treatment of cancer. We used shikonin to treat multi-resistant non-small lung cancer cells (DOC-resistant A549/D16, VCR-resistant A549/V16 cells) and defined the anti-cancer efficacy of shikonin. Our results showed shikonin induces apoptosis in these *ABCB1*-dependent and independent chemoresistance cancer sublines. Furthermore, we found that low doses of shikonin inhibit the proliferation of lung cancer stem-like cells by inhibiting spheroid formation. Concomitantly, the mRNA level and protein of stemness genes (*Nanog* and *Oct4*) were repressed significantly on both sublines. Shikonin reduces the phosphorylated Akt and p70s6k levels, indicating that the PI3K/Akt/mTOR signaling pathway is downregulated by shikonin. We further applied several signaling pathway inhibitors that have been used in anti-cancer clinical trials to test whether shikonin is suitable as a sensitizer for various signaling pathway inhibitors. In these experiments, we found that low doses shikonin and dual PI3K-mTOR inhibitor (BEZ235) have a synergistic effect that inhibits the spheroid formation from chemoresistant lung cancer sublines. Inhibiting the proliferation of lung cancer stem cells is believed to reduce the recurrence of lung cancer; therefore, shikonin’s anti-drug resistance and anti-cancer stem cell activities make it a highly interesting molecule for future combined lung cancer therapy.

## 1. Introduction

Non-small cell lung cancer (NSCLC) accounts for approximately 80% of lung cancer cases. Primary therapeutic strategies include surgery, chemotherapy, radiotherapy, target therapy, and immunotherapy. Unfortunately, the diagnosis is usually made at an advanced stage where the prognosis is poor and therapeutic options are limited. A stable 5-year survival rate of only about 15% has been estimated, despite advanced medical efforts in lung cancer therapy [1]. Since approximately 60% of NSCLC tumors do not harbor targetable driver mutations [2], conventional cytotoxic chemotherapy remains the standard-of-care treatment for NSCLC patients. The most common cytotoxic agents used in treating NSCLC patients are alkylating agents, including cisplatin and carboplatin, which damage DNA by forming platinum–DNA adducts and, thus, disrupt DNA replication and transcription [3]. However, tumor cells adapt mechanisms to protect against alkylating agents, which become obstacles to the successful treatment of NSCLC. Fortunately, several novel active agents for the treatment of lung cancer have been identified. Vinorelbine, gemcitabine (GEM), docetaxel (DOC), paclitaxel (PTX), and pemetrexed (PEM) are now used as chemotherapeutic agents that have shown promising activity, and their cisplatin combinations have further advanced survival for lung cancer patients [4]. However, the rapid development of resistance to these chemotherapeutic agents represents an important challenge to clinicians. Although these chemotherapies can initially reduce the tumor burden, relapse occurs in most cases. Drug resistance and recurrence are the two major causes of cancer death. Hence, there is an urgent need to identify safe and effective agents for use in chemoresistant lung cancer treatments.

The most common cause of cancer relapse and chemoresistance is attributed to the presence of cancer stem cells (CSCs) in tumor tissues [5]. CSCs represent a small population within the tumor mass that are responsible for aggressive tumor growth, metastasis, and therapy resistance. Similar to normal tissue stem cells, CSCs exhibit significant phenotypic and functional heterogeneity. While CSCs have been reported in a wide spectrum of human tumors, the biology of CSCs in NSCLC remains elusive [6]. Current anti-cancer therapies fail to eradicate CSC clones and instead favor the expansion of the CSC pool and select for resistant CSC clones, thereby resulting in treatment resistance and subsequent relapse in these patients. While multiple surface markers have been identified to date, with different profiles reported for different cancer types, the biology of lung CSCs and characterization of reliable surface markers remain elusive and warrant further investigation. CSCs in solid tumors are identified using an extensive list of markers. To date, a number of markers, which are expressed at the cell surface, intracellularly, have been identified in lung CSCs (CD133, CD166, CD44, CD90, CD87, Side population, and ALDH) [7]. ATP-binding cassette (ABC) transporters, such as p-glycoprotein (P-gp, *ABCB1*) and multidrug-resistant associated protein (MRP1), are membrane transporters that can pump structurally unrelated small molecules, such as cytotoxic chemotherapeutic drug, out of the cell. Normal stem cells and lung CSCs express high levels of ABC-transporters, resulting in low intracellular drug concentrations [8]. After CSCs are isolated, candidate CSCs are typically analyzed to see if they display the functional properties of CSCs. Transcription factors octamer-binding transcription factor 4 (Oct4), SRY-box transcription factor 2 (Sox2), and Nanog homeobox (Nanog) have been identified as core transcription factors that maintain embryonic stem cell self-renewal [9]. Under hypoxic conditions, Oct4 and Sox2 induced by hypoxia-inducible factor 1 alpha (HIF1α) and hypoxia-inducible factor 2 alpha (HIF2α) have been found to up-regulate the *CD133* promoter in NSCLC cell lines [10] and overexpression of Oct4 and Nanog in NSCLC cell lines have been shown to induce stem cell properties like self-renewal, tumorigenesis, invasion, and metastasis [11]. Therefore, the elimination of lung CSCs is of utmost importance at the time of therapeutic intervention in order to prevent CSCs expansion and subsequent tumor recurrence. 

Shikonin is a natural naphthoquinone pigment isolated from *Lithospermum erythrorhizon*. It has been reported to suppress the growth of various cancer cells such as breast (MCF-7) cells [12], gastric (AGS, AZ521, and SCM-1) cells [13], high-grade glioma (U87MG) cells [14], and lung cancer (A549) cells [15,16]. The anti-migration and anti-invasion activities of shikonin present through c-Met inhibition in EGFR mutated and highly c-Met expressive HCC827 lung cancer cells [17]. The conversion of glucose to lactic acid in the presence of oxygen is known as aerobic glycolysis or the “Warburg effect”. Increased aerobic glycolysis is uniquely observed in cancers [18]. Pyruvate kinase M2 (PKM2) is responsible for the Warburg effect [18,19]. Shikonin is a specific inhibitor that selectively inhibits PKM2 activity and induces cell death in PKM2, but not PKM1, expressing cells [20]. Therefore, shikonin may have the potential to control the growth of malignant cells through metabolic regulation. The question is, does shikonin itself result in drug resistance? It has been reported previously that after 18 months of treatment with different cell lines (K562, MCF-7, and a drug-resistant cell line K562/Adr), shikonin and its analogs are weak inducers of cancer drug resistance and can circumvent cancer drug resistance. These results indicate that shikonin is an incompetent inducer of cancer drug resistance [21]. Furthermore, to estimate the long-term systemic toxicity of shikonin derivatives (ShD) in a rat model, adult Wistar rats were gavage-fed with ShD at concentrations up to 800 mg/kg per day for 180 days. Hematological and biochemical examinations were performed, and the vital organs were subjected to pathological analyses. The investigators did not observe hematological or non-hematological toxicity of ShD at doses as high as 800 mg/kg per day for 6 months [22]. 

Deregulation of the PI3K/Akt/mTOR pathway is involved in lung tumorigenesis and it has been associated with advanced disease (stage III) [23]. Accordingly, inhibitors of the PI3K signaling pathway have been suggested as potential therapeutic agents in NSCLC. Since their catalytic domains are structurally similar, some second-generation mTOR kinase inhibitors have dual activity against both mTORC1-C2 and the p110 subunits of PI3K. These drugs, termed dual PI3K-mTOR inhibitors, have the potential of completely shutting down the PI3K/Akt/mTOR pathway, but this could result in greater toxicity and poor tolerance [24]. BEZ235, an orally available compound belonging to the class of imidazoquinolines, was the first PI3K inhibitor used in clinical trials in 2006. Unfortunately, in patients with advanced renal cell carcinoma, BEZ235 lacks efficacy and produces toxicities, and thus did not successfully proceed to further trial [25]. Similar results also have been reported by others in Japanese patients with advanced solid tumors [26] and advanced solid malignancies [27]. Increasing evidence has suggested that the application of BEZ235 in NSCLC may be possible by combination with other drugs in order to control drug resistance and reduce adverse effects [28,29]. 

In this study, the anti-proliferation effect of shikonin was investigated on chemoresistant NSCLC sublines. Although these chemoresistant sublines are capable of forming more sphere cells than their parental cancer cells, we treated these chemoresistant cancer cells with shikonin and examined by sphere-forming assay as well as evaluated the expression of stemness genes (*Nonog* and *Oct4*) to define the effect of shikonin anti-cancer activity. The signaling pathways associated with shikonin induction and apoptosis were analyzed. In order to improve drug tolerance and therapeutic efficacy, the possible drug–drug interaction of dual PI3K-mTOR inhibitor (BEZ235), JNK inhibitor (CC-930), and ERK inhibitor (LY3214996) with shikonin were further evaluated using a combination index (CI) and confirmed with sphere-forming assay.

## 2. Results

### 2.1. High Shikonin Sensitivity Was Observed from Parental A549 Lung Cancer and Its Chemoresistant Sublines

**Figure 1:**
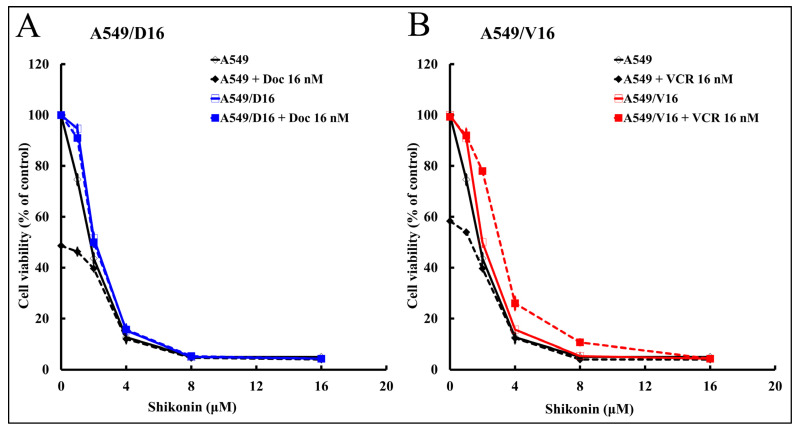
The sensitivities of lung cancer A549 cells and their chemoresistant sublines with shikonin were characterized by MTT cell survival assay. The sensitivities of A549 cells and their chemoresistant sublines A549/D16 (**A**) and A549/V16 (**B**) cells to shikonin (1, 2, 4, 8, and 16 μM) were compared when cells were treated with shikonin alone or combined with DOC (16 nM) or vincristine (VCR, 16 nM) accordingly for 48 h, followed by MTT assay.

To characterize whether shikonin has a growth inhibition effect on chemoresistant lung cancer cells, the parental A549 cells and their derived chemoresistant sublines, A549/D16 and A549/V16, were treated with shikonin alone or combined with DOC/VCR. The results in Figure 1A show that survival of A549/D16 cells was not reduced when they were treated with DOC (A549/D16+DOC 16 nM at 0 μM shikonin), thus confirming the existence of chemoresistance. In contrast, the survival of the parental A549 cells was inhibited by DOC to 50% (A549+DOC 16 nM at 0 μM shikonin) when compared to the mock control. Furthermore, low doses of shikonin (2 μM) significantly inhibited A549 and A549/D16 cell growth (Figure 1A). When shikonin was combined with DOC to treat A549/D16 cells, apparently, the survival profile was not affected when compared with cells treated with shikonin only. The data showed that both A549 and A549/D16 cells have similar shikonin sensitivities without DOC. We also examined the effect of shikonin on the A549/V16 subline (Figure 1B). Similar results were obtained, showing that low doses of shikonin (2 μM) significantly inhibit A549/V16 cell growth. Because the A549/V16 cells have P-gp independent drug resistance, shikonin not only inhibited P-gp dependent resistance on A549/D16 cells but also inhibited P-gp independent drug resistance of A549/V16 cells with a dose-dependent effect. According to these data, low doses of shikonin have shown the ability to overcome drug resistance in drug-resistant lung cancer cells without chemotherapeutic drugs. Therefore, shikonin has good potential to improve the therapeutic efficacy of chemoresistant lung cancer cells. The next question is, can shikonin inhibit the self-proliferation of stemness genes in lung cancer stem cells (CSCs)?

### 2.2. Shikonin Inhibits Spheroid Formation and Cancer Stemness Gene Expression

**Figure 2:**
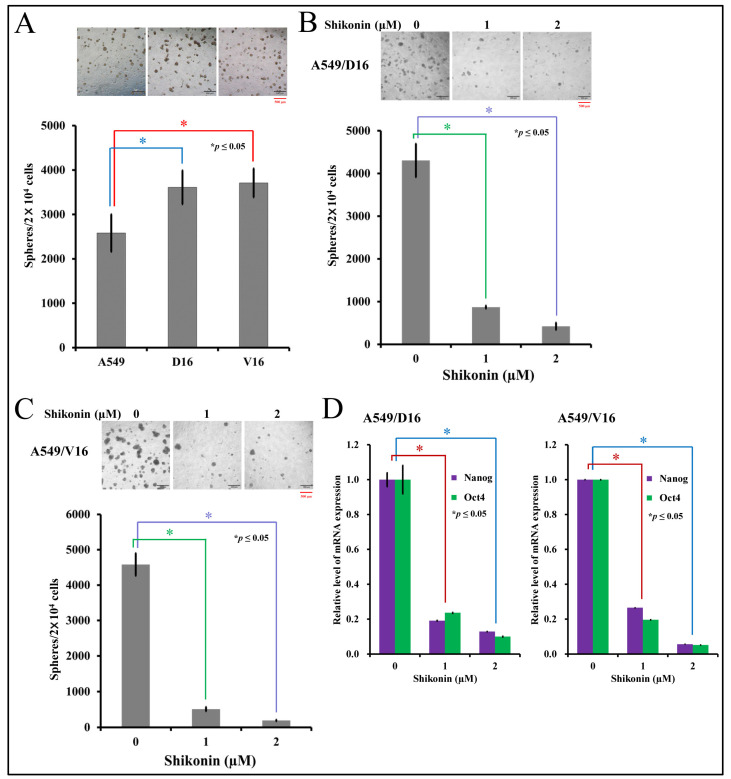
Characterization of shikonin-associated cancer stemness activities by sphere-forming assay and mRNA expression of stemness genes on chemoresistant lung cancer cells. The stemness of lung cancer and sublines were evaluated by the number of spheroids calculated from the secondary sphere-forming assay (**A**) followed by shikonin treatment on A549/D16 cells (**B**) and A549/V16 cells (**C**) at the beginning of the second assay with indicated concentrations. The spheroids were harvested for measuring Nanog and Oct4 expression by qRT-PCR (**D**). * *p* ≤ 0.05 is considered statistically significant (red scale bar length of 500 μm).

This study used the sphere-forming assay to determine the presence of CSCs in parental lung cancer A549 cells and chemoresistant sublines, and further compared their ability to form spheroid structure. After the secondary assay, the numbers of spheroids were calculated (Figure 2A), and the data showed that more spheroids were established from chemoresistant sublines. Next, after carrying out the primary sphere-forming assay, shikonin was added to the harvested cells for the secondary sphere-forming assay with A549/D16 cells (Figure 2B) or A549/V16 cells (Figure 2C). High levels of Nanog and Oct4 were expressed in the A549/D16 and A549/V16 sublines, but low doses of shikonin (1 μM) significantly inhibited spheroid formation. The expression level of Nanog and Oct4 mRNAs was concomitantly repressed on spheroids generated from both sublines when cells were treated with shikonin (Figure 2D), and the number of spheroids was reduced. The results suggest chemoresistance was associated with enhanced stemness activities in lung cancer cells. Furthermore, shikonin not only reduces the growth of lung cancer cells, it also strongly suppresses the stemness of chemoresistant lung cancer cells. 

### 2.3. Shikonin Induces Apoptosis of Chemoresistant Sublines and Reduces Phosphorylated Akt and p70s6k Levels with Downregulation of Stemness Genes 

**Figure 3:**
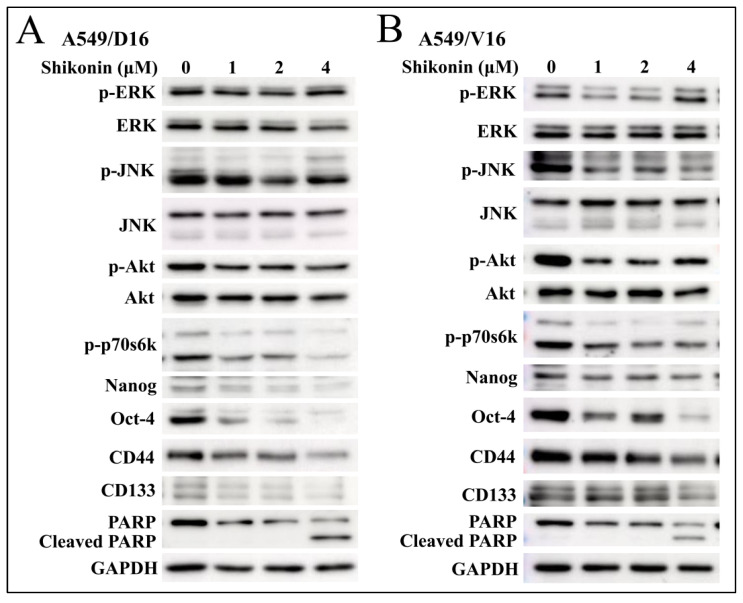
Evaluation of protein expression by Western blot analysis on shikonin-treated chemoresistant sublines. The signaling pathways of extracellular signal-regulated kinase (ERK), c-Jun N-terminal kinase (JNK), Akt and p70s6k were examined to determine the shikonin-regulated kinase on (**A**) A549/D16 and (**B**) A549/V16 cells. Meanwhile, the proteins of Nanog, Oct4, CD44, and CD133 affected by shikonin were also evaluated. The degradation of PARP into c-PARP indicates the presence of apoptotic cell death.

The signal pathways associated with shikonin anti-cancer effects in A549/D16 (Figure 3A) and A549/V16 (Figure 3B) chemoresistant lung cancer cells were examined by protein immune blots. The data showed that phosphorylation of Akt and p70s6k was significantly reduced with shikonin-dose dependence in both chemoresistant sublines. Furthermore, the expression of Oct4 and Nanog proteins was also coordinately downregulated when Akt was inhibited with 1 and 2 μM of shikonin treatment. Higher concentrations of shikonin (4 μM) apparently promoted poly (ADP-ribose) polymerase (PARP) degradation. These data suggest that apoptosis was coordinately established with high doses of shikonin treatment. Our data demonstrated the chemoresistance of A549 sublines, though they were selected from different chemotherapeutic drugs (DOC/VCR). Moreover, shikonin inhibits the same signal pathway to inhibit cell proliferation, and a higher concentration of shikonin induces apoptotic cell death. 

### 2.4. Low Concentrations of Shikonin Have a Synergistic Effect When Combined with PI3K-Akt-mTOR Inhibitor (BEZ235) to Reduce the Survival of Chemoresistant Lung Cancer Cells

**Figure 4:**
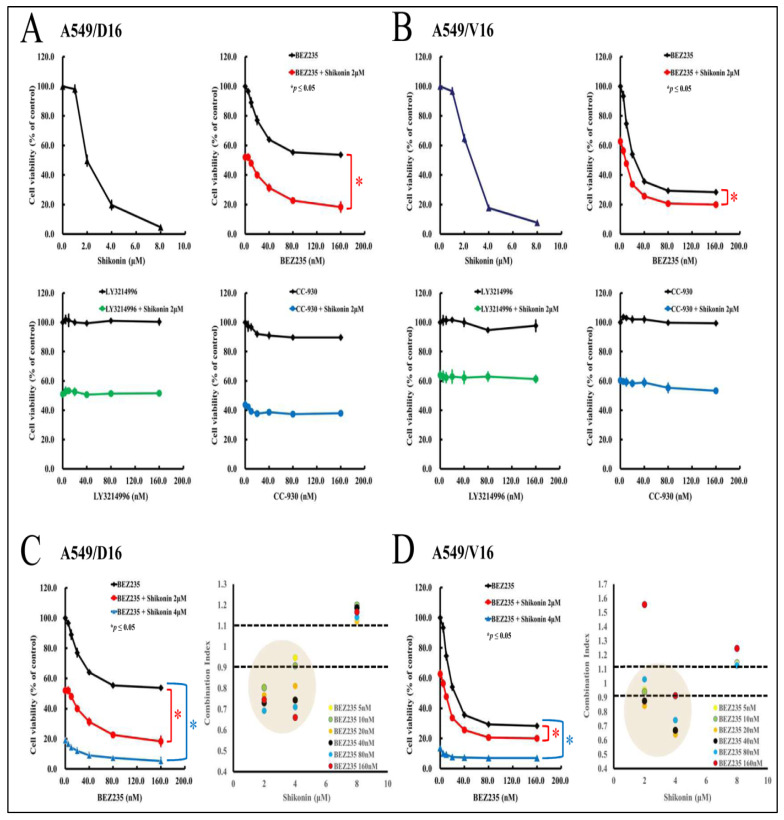
The drug–drug interaction of kinase inhibitors with shikonin on chemoresistant lung cancer cells. Firstly, the A549/D16 cells were used to determine their shikonin sensitivity. Other group of A549/D16 cells were pre-treated (1 h) with dual PI3K-mTOR inhibitor (BEZ235), ERK inhibitor (LY3214996), and JNK inhibitor (CC-930) with the indicated concentrations followed by shikonin (2 μM) with 48 h incubation. Then, the cell viability was determined by MTT assay (**A**). Similar experiments were applied to A549/V16 cells (**B**). The drug interactions were calculated for BEZ235 with shikonin (2, 4 μM) on A549/D16 (**C**) and A549/V16 cells (**D**) to analyze the interaction of BEZ235 and shikonin using a combination index (CI). * *p* ≤ 0.05 is considered statistically significant.

Although shikonin may inhibit the phosphorylation of JNK, Akt, and p70s6k, the exact signaling pathway remains to be further defined by comparing the effect of individual kinase inhibitors on cell viability. The A549/D16 cells were pre-treated (1 h) with the dual PI3K-mTOR inhibitor (BEZ235), ERK inhibitor (LY3214996), and JNK inhibitor (CC-930) with indicated concentrations, followed by treatment with shikonin (2 μM) for 48 h. Then, the cell viability was determined by MTT assay (Figure 4A). Our data showed that only BEZ235 can reduce cell viability, not LY3214996 and CC-930. These results confirm that the PI3K/Akt/mTOR pathway is essential for chemoresistant lung cancer A549 cell survival. Interestingly, when BEZ235 was combined with shikonin, the cellular viability was reduced significantly further (red line). Drug–drug interaction was not found when LY3214996 (green line) and CC-930 (blue line) were combined with shikonin. Similar results were also obtained from the A549/V16 cells (Figure 4B). The data demonstrated that the PI3K-mTOR inhibitor (BEZ235) enhances shikonin-mediated growth inhibition of chemoresistant cells in a dose-dependent manner. Therefore, we set up additional experiments to define the interaction between BEZ235 and shikonin by using a combination index (CI) model. According to this model, a quantitative analysis of synergism (CI < 1), additive effect (CI = 1), and antagonism (CI > 1) can be obtained. In case a more precise interpretation of data is required, an 11-point CI scale has been developed, starting from very strong synergism to very strong antagonism [30]. The CI data listed in Figure 4C,D suggest that a low concentration of BEZ235 (<80 nM) and shikonin (<4 μM) has a moderate synergism effect (0.7 < CI < 0.85) that represses the viability on chemoresistant lung cancer cells.

### 2.5. Combination of a Low Concentration of BEZ235 and Shikonin Significantly Reduced the Spheroid Formation on Chemoresistant Lung Cancer Cells

**Figure 5:**
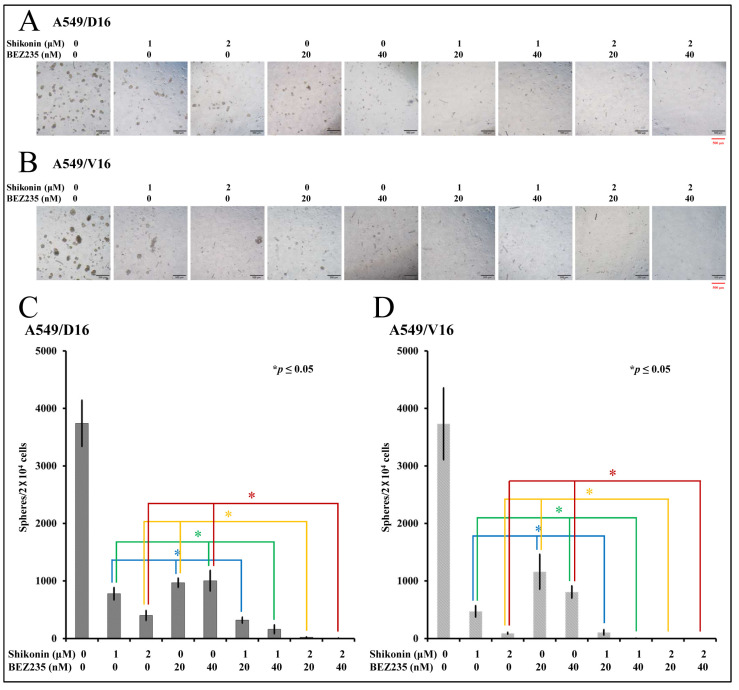
The evaluation of the anti-proliferation effect of BEZ235 and shikonin with sphere-forming assay on chemoresistant lung cancer cells. The number of spheroids calculated from the secondary sphere-forming assay with shikonin (0, 1, 2 μM) and/or BEZ235 (0, 20, 40 nM) treatment on A549/D16 cells (**A**,**C**) and A549/V16 cells (**B**,**D**) with indicated concentrations. * *p* ≤ 0.05 is considered statistically significant (red scale bar length of 500 μm).

To further evaluate the synergistic effect of BEZ235 and shikonin, the anti-CSC activities of BEZ235 combined with shikonin were demonstrated by sphere-formation assay. The A549/D16 cells harvested from the primary sphere-forming culture were treated with shikonin (0, 1, 2 μM), BEZ235 (0, 20, 40 nM), and their combination for the secondary sphere-formation assay, followed by photography with a microscope (Figure 5A). The numbers of spheroids were counted and listed in Figure 5C. The A549/V16 cells were also subjected to similar experiments; Figure 5B,D presents the findings. When cells were treated with BEZ235 (20 nM in column 4 Figure 5A,B), the numbers of spheroids were markedly reduced but could still be observed. By applying either 1 or 2 μM of shikonin, the remaining numbers of spheroids were further reduced with a significance of *p* ≤ 0.05 (Figure 5C,D, columns 6 and 8). A higher concentration of BER235 (40 nM in column 5) had a similar anti-proliferation effect when compared with its low concentration set. The addition of shikonin (1 or 2 μM) reduced spheroid formation significantly (column 7 and 9) in cells treated with 40 nM of BEZ235. The data showed that although BEZ235 leads to moderate anti-proliferation activity in chemoresistant lung CSC-like cells, the addition of shikonin may synergistically suppress CSC proliferation with a low-dose combination of these two molecules.

## 3. Discussion

Lung cancers are notorious and easily evolve to become resistant to most therapeutic agents. Cancer stem cells (CSCs) have been accepted as a significant cause of dormancy, drug resistance, recurrence, and metastasis of lung cancer [7]. Up-to-date, chemotherapy is still generally applied to most lung cancer patients as the first-line treatment, but the outcomes are restricted by the occurrence of drug resistance. We have established DOC and VCR multidrug-resistant lung cancer A549 cells [31] and observed chemoresistant cells with higher cancer stem-like properties than parental A549 cells by producing more spheroids. Since the molecules of DOC and VCR are all derived from plants, we tried to define other small molecules that have the potential to overcome DOC/VCR-induced chemoresistance. Our data showed that those chemoresistant lung cancer cells have dose-related sensitivity to shikonin (1 to 4 μM) without DOC or VCR co-treatment and a high dose of shikonin (4 μM), inducing apoptotic cell death.

Previous reports have demonstrated that shikonin can inhibit PI3K/Akt signaling in lung cancer A549 cells [32], afatinib-resistant NSCLC cells [33], and paclitaxel-resistance A549 cells [34]. A higher concentration of shikonin (>2 μM) induced cleavage of PAPR has been reported [32] that is associated with significant apoptosis. We also obtained similar results from this study of DOC/VCR-resistant A549 cells. Therefore, all acquired data highlight the essential role of the PI3K/Akt/mTOR pathway in lung cancer progression. Indeed, the significance of this signaling pathway in NSCLC has been rigorously discussed, with strong encouragement for the development of innovative designs using novel agents to improve patient outcomes [24]. Furthermore, the PI3K/Akt/mTOR pathway also plays an important role in CSC development and, thus, shows promise as a specific therapy target for cancer management [35]. 

In order to exclude other signaling pathways that may contribute to the anti-proliferation effect, we applied the dual PI3K-mTOR inhibitor (BEZ235), ERK inhibitor (LY3214996), and JNK inhibitor (CC-930) alone or combined with shikonin on chemoresistant cells. The results obtained from the cell proliferation assay suggested that only BEZ235 reduces the survival of chemoresistant cells. In contrast, downregulation of the MAPK pathway (JNK and ERK) has no significant anti-proliferation activity. Further, our data showed that shikonin and BEZ235 have different drug levels of potency and efficacy. Because potency refers to the range of doses over which a chemical produces increasing responses, thus, BEZ235, with nM concentration, is said to be more potent than shikonin, with a μM concentration. However, the maximal efficacy of BEZ235 is less than that of shikonin. Maximal efficacy reflects the limit of the dose-response relationship on the response axis to the used drug. To better evaluate the drug–drug interaction of shikonin and BEZ235, the CI index of shikonin and BEZ235 were determined. The CI data were between 0.7 and 0.85, indicating that a moderate synergistic effect could be detected at low concentrations of both drugs. A higher concentration of shikonin (4 μM) induced significant PARP degradation. These data suggest that apoptosis could be coordinately induced upon shikonin treatment. Previously, we used curcumin to characterize its anti-cancer effect on both DOC/VCR resistant A549 cells [36], and we demonstrated that ERK activation by curcumin was not associated with curcumin-induced apoptosis by using the ERK inhibitor U0126. Instead, curcumin induced strong activation of the p38 MAPK as a pro-death signal for apoptosis of chemoresistant A549 cells. Although shikonin and curcumin are both small, plant-derived molecules, we found that specific signaling pathways were involved in the apoptotic death of chemoresistant A549 cells. In sum, despite the selection of chemoresistant A549 cells by individual chemotherapeutic drugs, shikonin suppresses the activities of the PI3K/Akt/mTOR signal pathway to attenuate CSC proliferation, and may also induce apoptotic cell death. 

The anti-proliferation effect of shikonin on spheroid cells established from chemoresistant cells is associated with the significant reduction of the stemness genes of Oct4 and Nanog expression, both in mRNA and protein levels, with shikonin-dose dependent responsiveness. The anti-CSC effect of shikonin had not been determined until this investigation. Logically, the anti-CSC effect may be partly due to the inhibition of the PI3K/Akt/mTOR pathway that has been recognized previously by other researchers [35,37] for CSC development. Another anti-CSC effect may come from the downregulation of PKM2 by shikonin, which is a specific PKM2 inhibitor. It has been reported that the knockdown of PKM2 in CD44^+^ lung cancer stem-like cells by siRNA significantly reduces spheroid formation and increases their sensitivity to cisplatin and gamma-ray treatment [38]. In addition, when wild-type epidermal growth factor receptors (EGFR) containing A549 cells were treated with gefitinib, a tyrosine kinase inhibitor (TKI), the protein level of PKM2 was increased. Interestingly, the combination of shikonin and gefitinib exhibited a synergistic antitumor effect with downregulated PKM2 [39]. Although patients with wild-type EGFR tumors may be unable to benefit from the targeted therapy of EGFR-TKI, shikonin provides an alternative solution to enhance the antitumor effect of EGFR-TKI on wild-type EGFR NSCLC.

The anti-lung cancer effects of BEZ235 have been shown in several investigations. The human lung cancer cell lines of EPLC, HCC and H1339 were used and exposed to BEZ235 and/or cisplatin with effective sensitivity [40]. A panel of lung cancer cells has been tested, and the data showed that BEZ235 reduces cell proliferation in vitro regardless of their EGFR status and suppresses tumor growth in a mouse xenograft model using H1975 cells [41]. The combination of cisplatin and BEZ235 has also shown strong synergistic anti-proliferation effects on cisplatin-resistant A549 cells [28]. Therefore, effective lung cancer therapy can be designed in conjunction with BEZ235 and new therapeutic drugs to fight against PI3K-mTOR activities. According to our data and previous results, shikonin is a good candidate to be used in combination with BEZ235 to inhibit lung cancer progression effectively. However, shikonin is a small, hydrophobic molecule with poor solubility; apparently, the “first pass” effect and rapid clearance, thus, resulting in low oral bioavailability. To improve the bioavailability and enhance the in vivo efficacy of shikonin, many shikonin-embedded nanomedicines have been developed, including liposomes, polymeric micelles, and nanoparticles, which have been reviewed by Yan et al. [42]. Therefore, the combination of shikonin-embedded nanomedicines with BEZ235 for lung cancer therapy can be expected in the future. 

According to our results, different inhibitors targeting the PI3K-Akt-mTOR signal pathway could be a novel approach for the treatment of malignant lung cancer. Considering the low toxicity and high efficacy characteristics of shikonin, the PKM2-specific inhibitor has potential to overcome chemoresistant CSCs. Our investigation may lead to a new therapy application to control lung cancer malignancy.

## 4. Materials and Methods

### 4.1. Cell Lines

Docetaxel (DOC) and vincristine (VCR) were obtained from Sigma-Aldrich (St. Louis, MO, USA) to be used as selection agents to treat NSCLC cell lines of A549 (wild type p53) cells were purchased from ATCC (Manassas, VA, USA). The chemoresistant sublines were established, as mentioned [31]. Under low-dose exposure and long-term selection, individual surviving subclones of cancer cells were harvested for further higher-dose selection. Several stable drug-resistant subclones (A549/D16, A549/V16 cells) were successfully obtained as the materials for our investigation. For example, the A549/D16 subline can proliferate in the presence of 16 nM of DOC treatment. Although these sublines only represent part of drug resistance, they may offer the potential for cancer prognosis and potential therapies. The cross-resistance of each subline to other previously untreated chemotherapeutics can be observed. For example, the A549/D16 subline has only been selected by DOC but is still strongly resistant to VCR and doxorubicin [31]. 

### 4.2. Cytotoxicity Assay (MTT Assay)

Shikonin was purchased from ChemFaces (Wuhan, Hubei, China), and the chemosensitivity of DOC or VCR alone or combined with shikonin was determined using MTT colorimetric assay, as previously described [36]. Approximately 2 × 10^4^ cells per well were seeded onto 24-well plates. After 24 h incubation, the cells were exposed to various concentrations of DOC or VCR in a fresh medium for 48 h. At the end of the exposure period, the supernatant was removed, and cells were washed with PBS. Then, 300 mL MTT (1 mg/mL; Sigma-Aldrich, Saint Louis, MO, USA) was added to each well, and cells were incubated at 37 °C for 2.5 h. After the supernatant was removed and the cells were washed with PBS, 300 mL 2-propanol solution was added per well to dissolve the water-insoluble formazan salt. The plates were shaken at 70 rpm at room temperature for 10 min. Finally, the absorbance was measured at 570 nm using an SPECTRA max 340 PC ELISA plate reader (Molecular Devices, San Jose, CA, USA). Mean values were calculated from three independent experiments. Similar procedures were performed to carry out the combination of shikonin with individual kinase inhibitors to measure drug sensitivity on sublines. The PI3K-mTOR inhibitor (BEZ235) and JNK inhibitor (CC-930) were obtained from Cayman Chemical (Ann Arbor, MI, USA). ERK inhibitor (LY3214996) was purchased from Selleck Chemicals (Houston, TX, USA). Anti-Oct4 antibody was obtained from Sigma, while anti-CD 44 antibody was bought from Cell Signaling (Danvers, MA, USA).

### 4.3. Quantitative Reverse Transcription Real-Time Polymerase Chain Reaction (qRT-PCR)

To analyze gene expression, A549/D16 and A549/V16 sublines were harvested, and total RNA was isolated using TRIZOL (Invitrogen, Carlsbad, CA, USA). Reverse transcription was carried out by Applied Biosystems™ High-Capacity cDNA Reverse Transcription Kit (Waltham, MA, USA) according to the instructions. We performed Applied Biosystems™ PowerUp™ SYBR™ Green Master Mix for the expression of selected stemness genes, using an ABI 7500 thermal cycler (Applied Biosystems). The reactions were set as 95 °C, 2 min for enzyme activation. amplification of 40 cycles with denature at 95 °C, 15 s followed by anneal/extend at 60 °C, 1 min, then the reaction ended at 4 °C. For data analysis, SDS 2.2 software was used. Detection of PCR products was accomplished by measuring the emitting fluorescence (Rn) at the end of each reaction step (reaction cycles). The threshold cycle (Ct) corresponds to the cycle number required to detect a fluorescence signal above the baseline. We performed gene expression analysis using the comparative (2^–ΔΔCT^) Ct method. The extracted delta Ct values (which represents the expression normalized to the ribosomal 18S expression) were grouped according to the drug-resistant cell lines. Primers were obtained from MDBio, Inc. (New Taipei City, Taiwan). The primer sequences of Nanog-F and Nanog-R are CTCCAACATCCTGAACCTCAGC and CGTCACACCATTGCTATTCTTCG, respectively. Those of Oct4-F and Oct4-R are CCTGAAGCAGAAGAGGATCACC and AAAGCGGCAGATGGTCGTTTGG, respectively.

### 4.4. Primary and Secondary Sphere-Forming Assay

Cells (20,000 cells) were seeded in a non-adherent Nunclon Sphera Surface (Thermo Fisher Scientific, Waltham, MA, USA) and serum-free conditions with 0.4% BSA, EGF (20 ng/mL), bFGF (20 ng/mL), insulin (5 μg/mL), heparin (4 μg/mL), hydrocortisone (1 μg/mL) and B27 (1×) supplements were added to the DMEM/F12 medium. Adapted from a recently reported method [43], the numbers of primary spheroids were collected and measured on day 14 (first growths in medium). Then, the collected spheroids were dispersed and re-seeded with 20,000 cells again as a secondary assay (with triplicates in a six-well plate) for another 14 days, followed by the use of an inverted microscope photographed and counting. The numbers of spheres were counted by taking six fields per well as the total number of spheres. The final counted number served as the mean of the triplicated wells.

### 4.5. Protein Western Blot Assays

Proteins were reacted with one of the following antibodies for ERK, phospho-ERK (p-ERK), JNK, phospho-JNK (p-JNK), Akt, phospho-Akt (p-Akt), phospho-p70s6k (p-p70s6k), and poly (ADP-ribose) polymerase (PARP), which was obtained from Cell Signaling (Danvers, MA, USA), or GAPDH, which was purchased from Proteintech (Rosemont, IL, USA). The relevant procedures have been described in a previous report [36].

### 4.6. Statistical Analysis

All values are listed as mean ± SD. Data were compared among groups using a *t*-test, and * *p* < 0.05 is considered statistically significant. The combination index (CI) developed by Chou and Talalay is based on the median-effect equation for the non-linear dose-effect relationship [44]. 

## 5. Conclusions

In summary, the anti-proliferation effect of shikonin was demonstrated on chemoresistant A549 sublines. Shikonin significantly reduced spheroid formation and repressed the expression of stemness genes. The signaling pathway of PI3K/Akt/mTOR was downregulated by shikonin, and apoptotic cell death was detected. When cells were pre-treated with dual PI3K-mTOR inhibitor (BEZ235), JNK inhibitor, and ERK inhibitor, followed by shikonin treatment, only BEZ235 synergistically interacted with shikonin and led to a reduction in spheroid formation. In order to avoid cytotoxic effects from BEZ235 and control cancer recurrence, the application of low-dose BEZ235 with low-dose shikonin for NSCLC combination treatment may lead to an improvement in drug tolerance and therapeutic efficacy.

## Data Availability

The data used and/or analyzed during the current study are available from the corresponding author upon reasonable request.
